# Autoinducer production and quorum-sensing dependent phenotypes of *Pseudomonas aeruginosa *vary according to isolation site during colonization of intubated patients

**DOI:** 10.1186/1471-2180-7-33

**Published:** 2007-04-18

**Authors:** Sabine Favre-Bonté, Eric Chamot, Thilo Köhler, Jacques-A Romand, Christian van Delden

**Affiliations:** 1 Service des Maladies Infectieuses, Hôpital Universitaire de Genève, Rue Micheli-du- Crest 24, CH-1211 Genève 14, Switzerland; 2 Department of Epidemiology, University of Alabama, Birmingham, USA; 3 Service des Soins Intensifs, Hôpital Universitaire de Genève, Genève, Switzerland; 4 UMR CNRS 5557-Ecologie Microbienne, Université Claude Bernard, Lyon 1, France

## Abstract

**Background:**

*Pseudomonas aeruginosa *frequently colonizes and is responsible for severe ventilator-associated pneumonia in intubated patients. A quorum-sensing (QS) circuit, depending on the production of the two QS-signaling molecules (autoinducers, AIs) 3-oxo-C_12_-HSL and C_4_-HSL, regulates the production by *P. aeruginosa *of several virulence factors and is required for biofilm formation. Therefore QS-inhibition has been suggested as a new target for preventive and/or therapeutic strategies. However the precise role of QS during colonization and subsequent infections of intubated patients remains unclear.

**Results:**

We wondered whether QS is active during colonization of intubated patients, and whether *P. aeruginosa *isolates growing inside the biofilm covering the intubation devices and those resident in the lungs of colonized patients differ in their QS-dependent phenotypes. We collected the intubation devices of eight patients colonized by *P. aeruginosa*. We detected 3-oxo-C_12_-HSL on eight, and C_4_-HSL on six of these devices. In three of these patients we also obtained *P. aeruginosa *isolates from tracheal aspirates at the time of extubation (n = 18), as well as isolates from the intubation devices (n = 25). We genotyped these isolates, quantified their AIs production, and determined three QS-dependent phenotypes (adherence capacity, biofilm and elastase production). The production of 3-oxo-C_12_-HSL was consistently increased for isolates from the intubation devices, whereas the production of C_4_-HSL was significantly higher for isolates from tracheal aspirates. Isolates from tracheal aspirates produced significantly higher amounts of elastase but less biofilm, and had a marginally reduced adhesion capacity than isolates from the intubation devices. Levels of 3-oxo-C_12_-HSL and elastase production correlated statistically for tracheal intubation isolates, whereas levels of 3-oxo-C_12_-HSL production and adhesion ability, as well as biofilm production, correlated weakly amongst intubation device isolates.

**Conclusion:**

Our findings demonstrate that autoinducers are produced during the colonization of intubated patients by *P. aeruginosa*. The microenvironment, in which *P. aeruginosa *grows, may select for bacteria with different capacities to produce autoinducers and certain QS-dependent phenotypes. QS-inhibition might therefore affect differently isolates growing inside the biofilm covering intubation devices and those resident in the lungs.

## Background

*Pseudomonas aeruginosa *is an opportunistic pathogen implicated in a wide variety of infections, particularly in burn victims, cancer patients and cystic fibrosis (CF) patients. In addition *P. aeruginosa *is a major cause of severe pneumonia in mechanically ventilated patients [1]. Such ventilator-associated pneumonia (VAP) are almost always preceded by the colonization of the upper respiratory tract. The presence of the intubation device is a major risk factor for this colonization [2]. As such the capacities of *P. aeruginosa *to adhere to the inert surface and grow inside a biofilm on the intubation device are thought to be essential for the subsequent colonization and infection of the lower respiratory tract [3].

After colonizing the respiratory tract of intubated patients, *P. aeruginosa *causes infections and extensive damage to host tissues via the production of a number of extracellular virulence factors [4]. The expression of several of these factors is controlled by a quorum-sensing (QS) circuitry involving at least two autoinducers (AIs): N-(3-oxododecanoyl)-Homoserine Lactone (3-oxo-C_12_-HSL) and N-butyryl-L-Homoserine Lactone (C_4_-HSL) [4]. The production of 3-oxo-C_12_-HSL and C_4_-HSL requires the autoinducer synthases encoded by the *lasI *and *rhlI *genes, respectively. These QS-systems were first described for their role in the regulation of extracellular virulence factors [5,6] but have recently been shown to regulate many other bacterial functions including biofilm formation [7-10].

The importance of a functional QS-circuit for the virulence of *P. aeruginosa *has been clearly established *in vivo *in animal models [11]. As a consequence QS-inhibition has been suggested as a potential target for new preventive and/or therapeutic strategies of *P. aeruginosa *infections.

However the precise role during human infections remains speculative. Most studies so far have focused on the capacity of clinical isolates to produce the two autoinducers and on the detection of the two AIs directly in clinical samples. *P. aeruginosa *isolates from CF patients produce AIs *in vitro *[12], and sputum of CF patients colonized by *P. aeruginosa *produces the same signals *ex vivo *[13]. AIs have also been detected *in situ *in CF lung tissue [14], in the sputum of CF patients [15,16], and in bronchoalveolar lavage fluid after lung transplantation [17]. Recently we observed that most strains initially colonizing intubated patients are QS-proficient [18]. Both 3-oxo-C_12_-HSL and C_4_-HSL have also been detected in biofilms, both *in vivo *and *in situ *[15,16,19]; moreover their relative abundance has been suggested as a marker for the biofilm mode of growth in CF patients [13].

Because much remains to be understood about the role of QS during human infections, and the potential of QS-inhibition in their prevention and/or treatment, we examined the behavior of QS during the colonization of intubated patients. To determine whether QS is active during the colonization of intubated patients, we first extracted and quantified AIs directly from the intubation devices of patients colonized with *P.aeruginosa*. Because of the multitude of phenotypes depending on QS we then wondered whether *P. aeruginosa *isolates growing in different microenvironments might vary in their capacity to produce AIs and differ in their expression of QS-dependent phenotypes. We therefore collected *P. aeruginosa *isolates from the surface of intubation devices and from tracheal aspirates obtained from the same patients on the day of extubation. For each patient, we grouped strains according to their genotype, and characterized them in terms of production of autoinducers, and capacity to adhere to PVC, and produce elastase and biofilm. Our results suggest that the relative production of autoinducers, and the QS-dependent phenotypes of *P. aeruginosa *vary according to their site of isolation.

## Results

### Study population

Patients' clinical characteristics are presented in Table 1. Underlying diseases included polytrauma, intra-cerebral hemorrhage, congestive heart failure and non-pseudomonal sepsis. Duration of mechanical ventilation ranged from 9 to 24 days. Detailed clinical characteristics are provided for the three patients from whom *P. aeruginosa *isolates were collected both from the intubation device and from a tracheal aspirate on the day of extubation:

Patient 8 was a 27-year-old male who suffered a severe polytrauma including cerebral contusion and hemorrhage. *P. aeruginosa *was cultured for the first time from his tracheal aspirates 7 days after his intubation. Therapeutic retreat was decided after 15 days of mechanical ventilation and he died without having received any antibiotics. The presence of *P. aeruginosa *in his tracheal aspirates was considered as colonization. The tracheal aspirate used in the present study was collected shortly before his death and extubation.

Patient 12 was a 21-year-old female who suffered a severe polytrauma including a cranio-cerebral trauma with diffuse axonal lesions and multiple rib fractures. *P. aeruginosa *was first cultured in bronchoalveolar lavage fluid four days after her intubation. *P. aeruginosa *pneumonia was diagnosed two days later and she was treated with an imipenem – tobramycin combination therapy. The clinical response was satisfying and she was extubated 3 days later still on antimicrobial therapy. A tracheal aspirate was obtained on the day of extubation.

Patient 13 suffered severe congestive heart failure with a cardiac arrest. *P. aeruginosa *was first cultured from a tracheal aspirate three days after her intubation. *P. aeruginosa *pneumonia was treated with cefepime during 10 days starting 6 days after intubation. She remained colonized by *P. aeruginosa *and was extubated after 17 days of mechanical ventilation.

### *In situ *quantification of autoinducers on intubation devices

The cuff of each endotracheal intubation device was removed and autoinducers were extracted from the biofilm as described in *Materials and Methods*. 3-oxo-C_12_-HSL was detected on all cuffs, the amounts varying between 0.4 and more than 10 nmoles per cuff (Table 1). C_4_-HSL was detected on six of the eight devices tested and its amount varied between 17 and 300 nmoles per cuff (Table 1). The *in situ *isolation of autoinducers from biofilms of the majority of the intubation devices suggests that QS might play a role during colonization by *P. aeruginosa *of such devices. The fact that we detected C_4_-HSL only from 6 devices could result from the lower sensitivity of our C_4_-HSL bioassay (2 nmoles/cuff) compared to the one used to quantify 3-oxo-C_12_-HSL (0.01 nmoles/cuff).

### Collection of *P. aeruginosa *isolates colonizing the intubation devices and the lungs of intubated patients

For the three patients in whom we obtained tracheal aspirates (TA) at the time of extubation we also collected *P. aeruginosa *isolates directly from the cuff of the intubation device (ID) as described in Material and Methods. The complete collection consisted of 18 TA isolates and 25 ID isolates partitioned as shown in Table 2. Genotyping of these strains by RAPD identified 4 different genotypes (B, C, D and P). Genotypes C (3 isolates from patient 12 and 1 isolate from patient 13) and P (3 isolates from patient 13) were only found in tracheal aspirates and were therefore excluded from the analyses described in the following paragraphs, when not otherwise stated. The final collection therefore consisted of 11 TA and 25 ID isolates representing genotype B (8 TA and 21 ID isolates) and genotype D (3 TA and 4 ID isolates) (Table 2).

### Phenotypic characterization of TA and ID *P. aeruginosa *isolates

#### (i) *In vitro *autoinducer production

**Figure 1:**
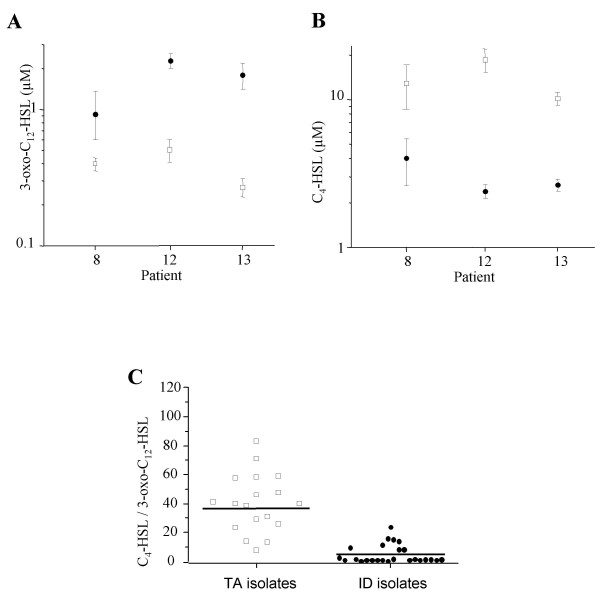
**Autoinducer production of *P. aeruginosa *isolates from tracheal aspirates and intubation devices**. 3-oxo-C_12_-HSL (A) and C_4_-HSL (B) production by *P. aeruginosa *isolates recovered from tracheal aspirates (open squares, TA isolates) and intubation devices (plain circles, ID isolates) of 3 colonized patients (8, 12 and 13). The production of the two autoinducers was determined in culture supernatants by specific bioassays. Error bars correspond to the standard errors of the means. (C) Ratios of AI concentrations (C_4_-HSL/3-oxo-C_12_-HSL) produced by 18 isolates recovered from tracheal aspirates and 25 isolates recovered from intubation devices. The horizontal line indicates the mean of the ratios.

We extracted and quantified the two autoinducers from culture supernatants of TA and ID isolates. All isolates produced both autoinducers *in vitro*, though levels varied according to the site of isolation (Table 2). Figure 1A and 1B show the mean production of both AIs for the isolates obtained from TA and ID for each individual patient. For the three patients ID isolates consistently produced higher amounts of 3-oxo-C_12_-HSL than TA isolates (Fig. 1A). When all the ID isolates were compared to the TA isolates the mean production of 3-oxo-C_12_-HSL was increased in the ID group (1.6 × 10^-6^M; Standard Error [SE], 4.8 × 10^-7^M), compared to the TA group (4.0 × 10^-7^M; SE, 7.4 × 10^-8^M, p = 0.12) without reaching statistical significance, possibly due to the small sample size. In contrast the TA isolates of all patients produced higher amounts of C_4_-HSL than the ID isolates (Fig. 1B), and the mean C_4_-HSL concentration was significantly higher for the TA isolates group (1.2 × 10^-5 ^M; SE, 1.7 × 10^-6^M) than for the ID isolates group (3.1 × 10^-6^M; SE, 5.4 × 10^-7^M; p = 0.04). Interestingly all isolates (including genotypes C and P), whatever their site of collection, produced higher levels of C_4_-HSL than of 3-oxo-C_12_-HSL. The mean of the C_4_-HSL/3-oxo-C_12_-HSL ratios was 38.5 for the TA group compared to 5.1 for the ID group (p = 0.002; Fig. 1C). Excluding the TA isolates with genotype C and P did not affect these results significantly. Therefore the relative amounts of AIs produced *in vitro *were significantly different between TA and ID isolates. The fact that all *P. aeruginosa *isolates tested were QS-proficient, i.e. able to produce both AIs *in vitro*, supports a role for QS during late stages of colonization of intubated patients both in the biofilm formed on intubation devices and in the lung.

#### (ii) Production of QS-dependent virulence factors

**Figure 2:**
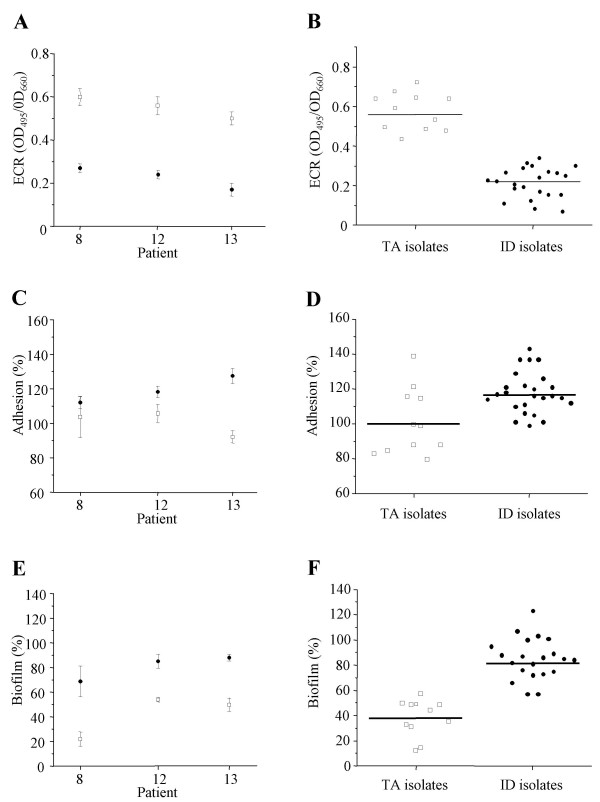
**Quorum-sensing dependent phenotypes of *P. aeruginosa *isolates from tracheal aspirates and intubation devices**. Elastase production (A and B), adhesion capacity (C and D) and biofilm formation (E and F) by *P. aeruginosa *isolates recovered from tracheal aspirates (open squares, TA isolates) and intubation devices (plain circles, ID isolates). Elastolytic activities were determined by ECR assays and expressed as the OD measured at 495 nm per unit of OD 660 nm. The adhesion capacities and biofilm formation were expressed as the percentage relative to the level obtained with the wild-type *P. aeruginosa *strain PT5. (A, C and E) Results individualized for each patient (8, 12 and 13), error bars correspond to the standard errors of the means. (B, D and F) Mean values and variances for 11 tracheal aspirate isolates and 25 isolates recovered from the intubation devices. For each individual isolate the determinations were performed twice in triplicates.

To further characterize the *P. aeruginosa *isolates, we determined their *in vitro *production of three virulence factors that depend upon an active QS-circuitry, namely, elastase, protease, and rhamnolipids [4]. We assayed the production of proteases and rhamnolipids using semi-quantitative plate assays. Important fluctuations in the production of rhamnolipids were observed (Table 2). This finding was not surprising as we had previously found fluctuations among QS-associated phenotypes occurring during the colonization of intubated patients [18]. LasB elastase is the most potent elastase produced by *P. aeruginosa *and is one of the major virulence factors controlled by QS [4]. We quantified the *in vitro *elastase production of the clinical isolates by means of the Elastin Congo Red assay at the same point in the growth curve (early stationary phase) as for determination of AIs production. For each patient the isolates obtained from the TA displayed higher elastolytic activities than those obtained from the ID (Fig. 2A). When the isolates were grouped by site of isolation, the mean elastase production was higher in the TA group (0.57; SE, 0.02) than in the ID group (0.23; SE, 0.02; p < 0.001; Fig. 2B). These results did not differ when all the genotypes were included in the TA isolates group. These results suggest that *P. aeruginosa *strains originating from the lungs of intubated patients produce higher amounts of elastase than isolates that were resident inside the biofilm on the intubation device.

#### (iii) Adhesion and biofilm formation capacity

QS has been suggested to be involved in the formation and differentiation of biofilms [7,8,13]. The impact of the type of surface and medium used in biofilm formation assays appears to influence the relationship between QS and biofilm formation [20]. We therefore decided to measure biofilm formation with our well-established static system using sterile PVC coupons obtained from intubation devices [8]. In our opinion this model simulates the best the conditions found on intubation devices in patients. Both adhesion and biofilm formation capacities were compared to the laboratory strain *P. aeruginosa *PT5 (100%). For each individual patient *P. aeruginosa *isolates recovered from the surface of the ID adhered more efficiently than their counterparts collected from the TA (Fig. 2C). When all the isolates were grouped by site of isolation the mean adhesion capacity was slightly increased in the ID group without reaching statistical significance (118.0%; SE, 4.0%) as compared to the TA group (101.3%; SE, 5.2%; p = 0.19; Fig. 2D). The ID isolates for each patient demonstrated a greater ability to form biofilm than their TA counterparts (Fig. 2E). This difference was statistically significant when the strains were grouped according to their site of isolation (ID isolates: 79.3%; SE, 6.3%, TA isolates: 39.1%; SE, 9.8% p= 0.01, Fig. 2F). Again these results were not affected when genotypes C and P were included in the TA group. These observations indicate that *P. aeruginosa *isolates originating from the biofilm of intubation devices tend to adhere more efficiently and have higher biofilm production capacities than their counterparts isolated from the lungs.

### Correlations between autoinducer production and quorum-sensing dependent phenotypes

#### (I) Correlations between autoinducer and elastase production

**Figure 3:**
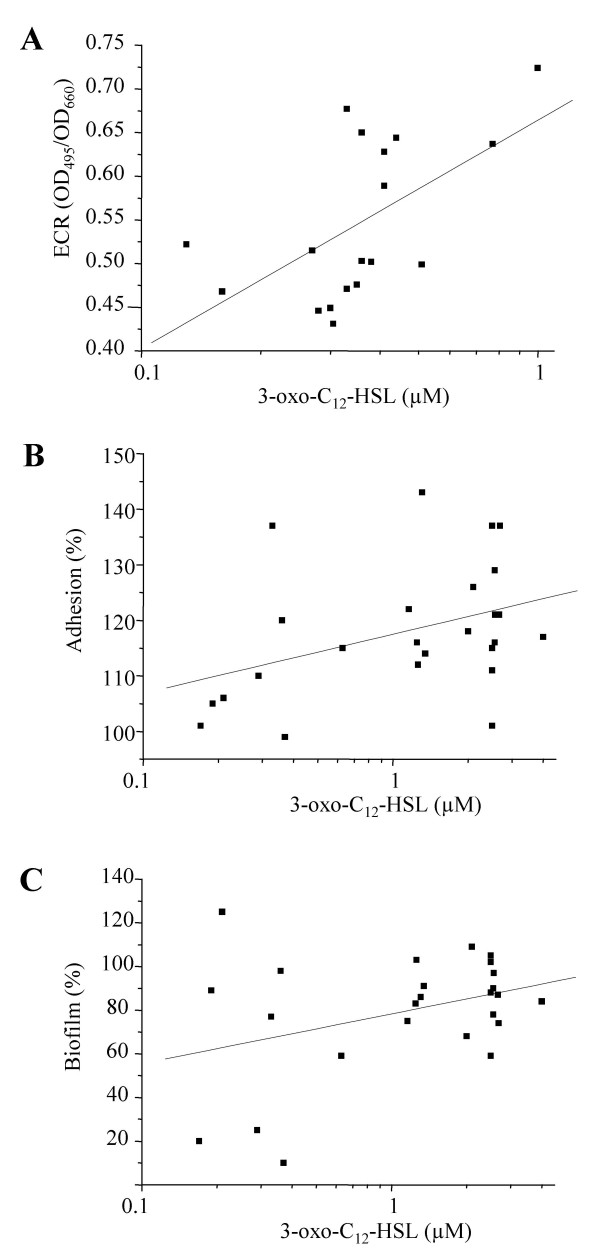
**Correlations between autoinducer production and quorum-sensing dependent phenotypes**. Relations between levels of 3-oxo-C_12_-HSL and elastase production *by P. aeruginosa *isolates recovered from tracheal aspirations (A), and adhesion capacity (B) as well as biofilm formation (C) by *P. aeruginosa *isolates recovered from intubation devices. (A) For each isolate the production of 3-oxo-C_12_-HSL and elastase was measured in aliquots taken at the same time during the growth curve, and plotted against each other. (B and C) For each isolate aliquots for 3-oxo-C_12_-HSL determinations were taken at an identical time during the growth curve. Adhesion capacities and biofilm formation are expressed compared to wild type strain PT5 (100%). Each determination is the mean of two independent experiments performed in triplicate.

*In vitro*, the production of elastase is under the joint control of both the *las *and *rhl *QS-systems [4,9]. In sputum of CF patients the expression of *lasR *has been correlated with the accumulation of *lasB *transcripts [21]. Other investigators have described a weak correlation between the *in vitro *transcription levels of *lasR *and *lasB *in 50% of environmental and clinical isolates [22]. We wondered whether the production of elastase would be correlated with the production of autoinducers. For these analyses we included only genotypes B and D, which were present in both TA and ID isolates. We observed a trend for a positive correlation between 3-oxo-C_12_-HSL and elastase production for the TA group (r = 0.58; p = 0.06). This positive correlation became statistically significant when genotypes C and P were included (r = 0.63, p = 0.006; Fig. 3A). In contrast for ID isolates we observed a statistically significant negative correlation between elastase and 3-oxo-C_12_-HSL production (r = -0.41, p = 0.04). Furthermore, C_4_-HSL production was not correlated with elastase activity, whatever the isolate group studied (TA isolates: r =-0.38, p = 0.25 and ID isolates: r = 0.02, p = 0.70). These results suggest that the level of 3-oxo-C_12_-HSL is positively correlated to elastase production for isolates from tracheal aspirates of colonized patients, but not for *P. aeruginosa *isolates from biofilms covering the intubation devices.

#### (II) Correlation between autoinducer production, adhesion and biofilm formation capacities

We then examined correlations between autoinducer production and adhesion capacity. There was a weak, statistically not significant, correlation between 3-oxo-C_12_-HSL production and adhesion capacity for isolates from the ID group (r = 0.30, p = 0.14) (Fig. 3B). No such correlation was found for isolates from the TA group (r = 0.33, p = 0.32), even when genotypes C and P were included in the analysis (r = 0.30, p = 0.22). We found no correlation between C_4_-HSL production and adhesion for both ID isolates (r = 0.0; p = 0.99) and TA isolates (n = 11: r = 0.34; p = 0.30, and n = 18: r = 0.16; p = 0.53).

Similarly we observed a weak, statistically not significant, correlation between 3-oxo-C_12_-HSL and biofilm production for isolates of the ID group (r = 0.33, p = 0.11, Fig. 3C), but not for those recovered from TA (r = 0.25, p = 0.46), even after inclusion of genotypes C and P in the analysis (r = 0.17, P = 0.49). There was no correlation between C_4_-HSL and biofilm production for the ID (r = -0.18, p = 0.31), and TA isolates (r = -0.18, p = 0.60), despite the inclusion of genotypes C and P (r = 0.16, p = 0.52). Together these observations suggest that the production of 3-oxo-C_12_-HSL but not of C_4_-HSL, might be associated with *in vitro *adherence and biofilm formation capacities among isolates recovered from the biofilm on intubation devices.

## Discussion

Respiratory tract colonization of intubated patients by *P. aeruginosa *is initiated by the colonization of the intubation device by bacteria either originating from the digestive flora of the patient (endogenous acquisition) or transmitted via handling of the device by health care workers (exogenous acquisition) [23]. This colonization requires efficient bacterial adherence to the inert surface and the formation of a biofilm [3]. *P. aeruginosa *then reach the respiratory tract either by leakage around the cuff of the intubation device, by shearing of secretions containing embedded bacteria from the device during the inspiratory air flow, or by contamination and direct inoculation of the respiratory tract by suction tubes introduced through the intubation device by health care workers [23-25].

Both *in vitro *studies and animal models have suggested that QS plays a major role in the virulence of *P. aeruginosa *[11]. However *in situ *and in patient data concerning the potential role of QS during colonization and infection by *P. aeruginosa *in humans remain scarce. QS-activity has been linked to the detection of *P. aeruginosa *AIs in the lungs of cystic fibrosis patients [13-16], as well as in lung transplanted patients [17]. So far studies have only shown that most of the isolates originating from colonized intubated patients are QS-proficient [18,22].

In the present study we show that the two *P. aeruginosa *autoinducers, 3-oxo-C_12_-HSL and C_4_-HSL, can be detected *in situ *in biofilms covering intubation devices retrieved from patients colonized by *P. aeruginosa*. Moreover all *P. aeruginosa *isolates collected either from these biofilms or from tracheal aspirates produced these AIs *in vitro*. Whereas 3-oxo-C_12_-HSL has been previously shown to play a role in the differentiation of *P. aeruginosa *biofilms [7,26], C_4_-HSL seems to be important during the maturation stage of biofilm development [27], for the total amount of biofilm formed [8], and for the maintenance of biofilm architecture [28]. In our study, isolates recovered from the biofilm of intubation devices produced higher levels of C_4_-HSL than 3-oxo-C_12_-HSL *in vitro*. Previous observations have suggested that C_4_-HSL is produced in higher quantities than 3-oxo-C_12_-HSL in *P. aeruginosa *biofilms in the lungs of CF patients [13,14]. Singh et al. have even suggested that this AI ratio might serve as a biomarker for the biofilm mode of growth, as the mucoid isolates from the sputum of CF patients inverted their AI ratio when sub-cultured in liquid medium [13]. In contrast, in our study a higher level of C_4_-HSL as compared to 3-oxo-C_12_-HSL was observed when isolates were grown in liquid cultures, suggesting that this ratio is not useful as a biomarker for biofilm growth for these isolates. None of the isolates collected from our patients had a mucoid phenotype. This discrepancy could therefore be explained by inherent differences in biofilm development patterns between mucoid and non-mucoid strains. So far there is no evidence that non-mucoid *P. aeruginosa *colonizing the lung of intubated patients grows in biofilms. Such a hypothesis deserves further investigations.

Autoinducers have been previously recovered from biofilms formed on urinary tract catheters [19] and our results show for the first time that QS-signaling molecules are also produced in biofilms covering intubation devices. Whether the local concentrations of AIs on the intubation devices that we measured are physiologically relevant and are sufficient to activate the expression of QS-dependent genes remains speculative. However microarray analyses have shown that QS is a continuum and that the expression of some genes is already activated even at low AI concentrations [9]. It is therefore likely that even low concentrations of AIs on the intubation devices are physiologically relevant.

Intriguingly, the *in vitro *production of both AIs varied with the site of collection. Isolates from tracheal aspirates produced higher levels of C_4_-HSL but lower levels of 3-oxo-C_12_-HSL than their genotypically identical counter-parts isolated from intubation devices, even after several *in vitro *passages. This observation suggests that different microenvironments select *P. aeruginosa *isolates with various AI production capacities. Moreover the production of elastase, and the capacity to adhere to an inert surface and to produce a biofilm also varied with the site of isolation. Tracheal aspirate isolates produced higher levels of elastase, but were less able to adhere and produce biofilm than their counter-parts recovered from intubation devices. LasB elastase is one of the major virulence factors controlled by the *P. aeruginosa *QS-circuit [4]. *In vitro *its production is regulated by the QS-circuit and depends mainly on the production of 3-oxo-C_12_-HSL [9,10]. LasB elastase is believed to allow *P. aeruginosa *to invade surrounding tissues by its broad range enzymatic activity and to facilitate blood stream invasion by degradation of elastin fibers in the lamina propria of blood vessels [4].

The observed differences in phenotypes between isolates obtained from tracheal aspirates, compared to those from the intubation devices, make sense. Isolates growing in the biofilm on intubation devices might not invest energy in producing elastase, but might be primed to adhere and form biofilms, whereas isolates growing in the lungs might find an advantage in producing elastase. For tracheal aspirate isolates, levels of 3-oxo-C_12_-HSL production correlated positively with the capacity to produce elastase. In contrast for isolates originating from the intubation device, 3-oxo-C_12_-HSL levels did not correlate with elastase production, but correlated weakly with the capacity to adhere and to form biofilms on inert surfaces. These observations suggest that the previously described control of LasR on elastase production might not apply to all isolates depending on the site they were collected from. It seems that the microenvironment on the intubation devices selects for phenotypes that produce high levels of 3-oxo-C_12_-HSL but without concomitant increased elastase production. In contrast the lower lung environment seems to select for isolates that produce less 3-oxo-C_12_-HSL but with a positive control of this AI on elastase production. Two of our patients (12 and 13) received antimicrobial therapies during their mechanical ventilation. We can therefore not exclude that different concentrations of these drugs at the two sites might have selected different phenotypes. However this seems unlikely as isolates of patient 8 presented the same differences in phenotype in the absence of any exposure to antimicrobial therapies.

We detected no correlations between the levels of C_4_-HSL production and elastase activity, adhesion or biofilm formation. Strikingly, these phenotypes remained stable for each isolate independently of its origin even after several *in vitro *passages. This suggests that mutations in specific regulators, that affect the production of AIs and the expression of QS-dependent phenotypes might be selected in particular microenvironments. Indeed *lasR *and *rhlR *mutants of *P. aeruginosa *strains isolated from intubated patients have been previously described [18]. Such mutations influence the bacteria capacity to produce AIs, and are expected to affect QS-dependent phenotypes. We therefore sequenced both *lasR *and *rhR *in 6 TA and 6 ID isolates (Table 2). None of them harbored mutations compared to the wild type strain PAO1 (data not shown). This explains why all the isolates analyzed in the present study were QS-proficient. Phenotypic variations not linked to QS but also influencing biofilm formation have been described in *P. aeruginosa *isolates from CF patients [29]. The nature of the mutational events possibly selected either during biofilm growth on intubation devices, or inside the lung are presently under investigation. The identification of these events would have major implications for our understanding of bacterial selection in specific environments and the dynamics of bacterial populations.

QS-inhibition has been suggested as a new therapeutical approach against *P. aeruginosa *infections. For instance macrolides interfere with the production of AIs [8,30] and were shown to retard biofilm formation [31]. Our result might be important if QS-inhibitors that interfere with the production and/or activity of one, or both, of the autoinducers are used in the prevention or treatment of *P. aeruginosa *VAP. Indeed, our data suggest that inhibition of QS might have different effects on *P. aeruginosa *isolates depending on their microenvironnement.

## Conclusion

Our study was limited to the analysis of three patients; therefore it is difficult to draw general conclusions from our observations. However the results were consistent between these patients and reached statistical significance despite the small number. Our results showed that during colonization of intubated patients *P. aeruginosa *autoinducers are produced in the biofilms covering the intubation devices. The capacity to produce autoinducers and the expression of QS-dependent phenotypes vary with the site of isolation. Further studies will be required to investigate the mechanism that led to selection of specific phenotypes in a particular microenvironment directly *in vivo *and to determine how this impacts the use of anti-quorum-sensing strategies.

## Methods

### Patient population and clinical sample collection

We collected the intubation devices of eight patients known to be colonized by *P. aeruginosa *and hospitalized in the surgical intensive care service of the University Hospital Geneva. All patients, except patient 14, have been described previously (Table 1) [18]. Following extubation the endotracheal tubes were wrapped in a sterile cloth and transported without delay to the laboratory were they were stored at -70°C until use. Sterile cotton swabs were used to detach bacteria embedded in biofilm formed on the cuff, on the exterior surface of the intubation device (ID). *Pseudomonas *isolates were identified on selective Cetrimide agar plates. In order to avoid compiling identical *P. aeruginosa *isolates, we collected individual clones based on morphologic differences of their respective colony forming units (CFU). A total of 25 ID isolates were collected from patients 8, 12 and 13, and stored at -70°C (Table 2).

We obtained tracheal aspirates by suctioning secretions with a sterile tube introduced deeply inside the lower respiratory tract through the intubation device from three patients (patients 8, 12 and 13) either on the day before, or on the day of extubation. The tracheal aspirates (TA) were plated on selective agar plates (Cetrimide 0.03%) in order to identify *Pseudomonas *isolates [14]. Similarly to ID isolates, special care was taken to select isolates with different morphologic phenotypes according to their CFUs. A total of 18 different TA isolates were identified and stored at -70°C (Table 2).

For further assays both ID and TA isolates were taken from the frozen stocks and grown in liquid cultures.

### Media and culture conditions

Cultures for extraction of cell-to-cell signaling molecules and production of elastase were grown in Luria-Bertani (LB) broth. M63 medium [32] was used for bacterial adhesion assays and AP medium [33] was used for biofilm formation assays. No differences in growth rates were observed between the strains in these media.

### Extraction and quantification of acylated homoserine lactones (AIs) from intubation devices and liquid cultures

Previous studies have suggested that secretions colonized by bacteria accumulate above the cuff of intubation devices and form biofilms [34-36]. Leakage of bacteria around the cuff might play a major role in the development of ventilator-associated pneumonia [25,37]. Consequently, we selected the cuff of the intubation devices for the quantification of AIs. To extract AIs; cuffs were aseptically cut off the intubation devices, incubated for 30 minutes at 37°C in 5 ml sterile saline containing 0.025% dithiothreitol (DTT) to resuspend the biofilms formed on the cuff, and extracted twice by the addition of 1 volume of acidified ethyl acetate for 5 minutes at -20°C. To extract AIs from liquid cultures of both ID and TA isolates, the strains were incubated in LB broth at 37°C with shaking. Aliquots of filtered culture supernatants, taken after 8 hours of growth (early stationary phase), were subjected to 2 extractions in 1 volume of acidified ethyl acetate and stored at -70°C. Aliquots of the extracts obtained from the cuffs and liquid cultures of ID and TA isolates were evaporated under nitrogen gas and the concentrations of 3-oxo-C_12_-HSL and C_4_-HSL determined using the reporter strains *E. coli *MGλ1,4 (pPCS1) [38] and *P.aeruginosa *PAO-JP2 (pECP61.5) [39] respectively. For each determination standard curves were obtained with the wild type strain PAO1. The amount of AIs recovered from the intubation devices was expressed as nmoles/cuff.

### Determination of bacterial genotype

Bacterial strains were genotyped by Random Amplified Polymorphic DNA (RAPD) analysis as previously described using the Ready-to-Go Kit (Amersham) [18] using primer 208 (ACGGCCGACC) [40].

### Production of protease, rhamnolipids and elastase

Total extracellular protease [41] and rhamnolipid [42] production of both ID and TA isolates were determined semi-quantitatively by agar plate assays. Elastase production was quantified using the Elastin Congo Red elastolysis assay [38,41]. Culture supernatants were collected at the same point in the bacterial growth curve as for AI extraction.

### Assessment of bacterial adhesion and biofilm formation assay

Adhesion capacity was measured for both ID and TA isolates as the ability of bacteria to adhere to the wells of 96-well microtiter plates. The assay was performed as previously described [18]. Results were expressed as percent adhesion with respect to the PT5 wild type strain. The ability of bacteria to form biofilm *in vitro *was determined in a static model as previously described [8]. Briefly, overnight LB cultures of ID and TA isolates were diluted to an OD_660 _of 0.5 and incubated for a further 6 hours at 37°C with shaking until cells had entered early stationary phase. Sterile calibrated coupons of PVC tracheal tubing were immersed in the suspension and incubated statically at 37°C for 1 h, then the PVC coupons were transferred to 6-well cell culture plates and incubated statically at 37°C for 72 h in AP medium. The extent of biofilm formation was measured using crystal violet staining [43]. Results were expressed as percent biofilm formation with respect to the PAO1 wild type strain.

### Statistical analysis

We used the Student's t test to compare mean production of AIs and virulence factors by isolates from tracheal aspirates (TA) and the cuff of the intubation device (ID). Correlations between AIs production and QS-dependent phenotypes were calculated using a robust estimator of the Pearson's correlation coefficient. To account for the correlation among multiple observations in the same patient, standard errors and p values were computed using a first-order Taylor-series linearization method. All analyses were conducted using Stata 6.0 statistical software (StataCorp, 1999). p < 0.05 was considered statistically significant.

## Authors' contributions

SFB carried out the experiments and drafted the manuscript. CE conducted the statistical analysis. TK participated in the design of the study and participated in the analysis of the results. JAR organized the sample collection. CVD conceived the study, supervised the experiments and their analysis, and wrote the final manuscript. All authors read and approved the final manuscript.

## Figures and Tables

**Table 1:** Patient characteristics and intubation devices

Patient	Underlying disease	Duration of intubation (days)	Tracheal aspirate on day of extubation	3-oxo-C_12_-HSL (nmoles/cuff)	C_4_-HSL (nmoles/cuff)
2	Polytrauma	9	no	0.4	100
5	Nonpseudomonal sepsis	13	no	0.5	17
7	Intra-cerebral hemorrhage	24	no	> 10	22
8	Polytrauma	15	yes	> 10	22
11	Polytrauma	18	no	> 10	300
12	Polytrauma	9	yes	> 10	2
13	Congestive heart failure	17	yes	> 10	2
14	Polytrauma	16	no	6	24

**Table 2:** Characterization of *P. aeruginosa *isolates from intubated patients

Isolates	Patients	Genotype	Site of Isolation	Production^c ^of		Elastase^a^(OD_495_/OD_660_)	Production^a ^of		Adhesion^a ^(%)	Biofilm^a ^(%)
								
				Proteases	Rhamnolipids		3-oxo-C_12_-HSL (μM)	C_4_-HSL (μM)		
61G3^d^	8	D	TA	+ + +	BD^b^	0.5	0.51	15	88	31
61E8	8	D	TA	+ + +	BD	0.59	0.41	24	88	11
60A3^d^	8	B	TA	+ + +	+	0.68	0.33	7.8	100	13
60B5	8	D	TA	+ + +	BD	0.65	0.36	5	139	33
										
63E8	8	B	ID	+ + +	+	0.3	1.3	2.4	116	83
63E9	8	B	ID	+ + +	+ +	0.34	2.5	1.1	111	105
63E11^d^	8	B	ID	+ + +	+ + +	0.29	0.19	2.9	105	89
63G3	8	B	ID	+ + +	+	0.24	0.63	15	115	59
63G6	8	D	ID	+ + +	BD	0.3	0.36	3	120	98
63G8	8	B	ID	+ + +	+ +	0.2	0.21	3.3	106	125
63H2	8	B	ID	+ + +	+ + +	0.12	2.7	2.9	137	74
62F2	8	D	ID	+ + +	BD	0.32	0.37	3.1	99	10
62F3^d^	8	D	ID	+ + +	+	0.22	0.17	2.4	101	20
62G3	8	D	ID	+ + +	BD	0.3	0.29	3.3	110	25
										
54A7	12	C	TA	+ + +	+ +	0.5	0.36	30	104	59
54A8	12	B	TA	+ + +	+	0.63	0.41	19	115	49
54A9	12	C	TA	+ + +	+ + +	0.5	0.38	12	105	49
54B1	12	C	TA	+ + +	+	0.64	0.77	30	99	60
54D5^d^	12	B	TA	+ + +	+	0.48	0.35	14	116	50
54D6^d^	12	B	TA	+ + +	+ + +	0.43	0.31	18	80	53
54E1	12	B	TA	+ + +	+ +	0.72	1	7.9	121	58
										
55A1^d^	12	B	ID	+ +	BD	0.19	2.6	2.1	116	78
55A2	12	B	ID	+ +	+	0.27	1.2	1.3	122	75
55A5	12	B	ID	+ +	+ +	0.26	2.5	2.8	137	102
55D2	12	B	ID	+ +	+	0.27	2	3.4	118	68
55E4	12	B	ID	+ +	+ +	0.31	2.5	2.7	115	88
55F3^d^	12	B	ID	+ +	+ + +	0.22	2.5	2.2	101	59
55G2	12	B	ID	+ +	+ +	0.17	4	2	117	84
55G4	12	B	ID	+ +	+	0.19	1.3	1.6	112	103
55A6	12	B	ID	+ +	+ + +	0.25	2.1	3.4	126	109
										
56A1	13	B	TA	+ + +	+ +	0.64	0.44	6.4	85	46
59B3	13	C	TA	+ + +	+ +	0.45	0.3	7.9	88	39
59B4^d^	13	B	TA	+ + +	+ +	0.47	0.16	9.5	83	48
59A1^d^	13	B	TA	+ + +	+ + +	0.52	0.13	9.3	99	38
59B6	13	P	TA	+ + +	+ +	0.45	0.28	13.5	83	41
59C1	13	P	TA	+ + +	+ + +	0.52	0.27	11.3	106	77
59C2	13	P	TA	+ + +	+	0.47	0.33	13.3	101	59
										
63A1	13	B	ID	+ + +	+ +	0.23	1.4	1.8	114	91
63A3^d^	13	B	ID	+ + +	+ + +	0.3	0.33	3.1	137	77
61A12^d^	13	B	ID	+ + +	+ + +	0.15	2.6	2.2	129	90
61G12	13	B	ID	+ + +	+ +	0.11	2.7	2.4	121	87
62C2	13	B	ID	+ + +	+ +	0.15	2.6	3.1	121	97
62C3	13	B	ID	+ + +	+ + +	0.08	1.3	3.3	143	86

^a ^Values are mean of three independent experiments with a standard deviation of < 10%.

^b ^BD, below detection.

^c ^+++, high level; ++, intermediate level; +, low level.

^d ^*lasR *and *rhlR *genes sequenced
